# Pain Management in Cancer Center Inpatients: A Cluster Randomized Trial to Evaluate a Systematic Integrated Approach—The Edinburgh Pain Assessment and Management Tool

**DOI:** 10.1200/JCO.2017.76.1825

**Published:** 2018-03-15

**Authors:** Marie Fallon, Jane Walker, Lesley Colvin, Aryelly Rodriguez, Gordon Murray, Michael Sharpe, for the Edinburgh Pain Assessment and Management Tool Study Group

**Affiliations:** Marie Fallon and Lesley Colvin, University of Edinburgh, Institute of Genetics and Molecular Medicine, Edinburgh Cancer Research Centre; Aryelly Rodriguez and Gordon Murray, University of Edinburgh, Usher Institute of Population Health Sciences and Informatics, Edinburgh; and Jane Walker and Michael Sharpe, University of Oxford, Warneford Hospital, Oxford, United Kingdom.

## Abstract

**Purpose:**

Pain is suboptimally managed in patients with cancer. We aimed to compare the effect of a policy of adding a clinician-delivered bedside pain assessment and management tool (Edinburgh Pain Assessment and management Tool [EPAT]) to usual care (UC) versus UC alone on pain outcomes.

**Patients and Methods:**

In a two-arm, parallel group, cluster randomized (1:1) trial, we observed pain outcomes in 19 cancer centers in the United Kingdom and then randomly assigned the centers to either implement EPAT or to continue UC. The primary outcome was change in the percentage of study participants in each center with a clinically significant (≥ 2 point) improvement in worst pain (using the Brief Pain Inventory Short Form) from admission to 3 to 5 days after admission. Secondary outcomes included quality of analgesic prescribing and opioid-related adverse effects.

**Results:**

Ten centers were randomly assigned to EPAT, and nine were assigned to UC. We enrolled 1,921 patients and obtained outcome data from 93% (n = 1,795). Participants (mean age, 60 years; 49% women) had a variety of cancer types. For centers randomly assigned to EPAT, the percentage of participants with a clinically significant improvement in worst pain increased from 47.7% to 54.1%, and for those randomly assigned to continue UC, this percentage decreased from 50.6% to 46.4%. The absolute difference was 10.7% (95% CI, 0.2% to 21.1%; *P* = .046) and it increased to 15.4% (95% CI, 5.8% to 25.0%; *P* = .004) when two centers that failed to implement EPAT were excluded. EPAT centers had greater improvements in prescribing practice and in the Brief Pain Inventory Short Form pain subscale score. Other pain and distress outcomes and opioid adverse effects did not differ between EPAT and UC.

**Conclusion:**

A systematic integrated approach improves pain outcomes for inpatients in cancer centers without increasing opioid adverse effects.

## INTRODUCTION

Half of patients with cancer suffer from pain.^1-3^ Despite the availability of pain management guidelines, poor outcomes are common.^4-10^ Common shortcomings in pain management include unstructured assessment, use of treatment guidelines that lack explicit algorithms and do not address clinicians’ concerns about prescribing opioids, and lack of systematic monitoring of outcomes, including adverse effects.^11,12^

Admission to a cancer center provides an important opportunity to improve pain outcomes. We developed a simple clinician-administered bedside tool—the Edinburgh Pain Assessment and management Tool (EPAT)—that builds on the concept of pain as the fifth vital sign.^13-16^ EPAT aims to change routine practice by directing a systematic assessment of cancer-related pain, guiding treatment using linked algorithms, and prompting the regular reassessment of pain to determine both efficacy and adverse effects of treatment. We tested EPAT in a single cancer center and found preliminary evidence of its feasibility and efficacy.^16^

The current study aimed to determine the effectiveness of implementing EPAT in multiple cancer center inpatient units. Specifically, we sought to determine whether the percentage of inpatients with improved pain increased in cancer centers that implemented EPAT compared with cancer centers that continued to deliver usual care (UC). We also aimed to find out whether implementing EPAT improved prescribing practice and whether it increased opioid-related adverse effects.

## PATIENTS AND METHODS

### Study Design and Patients

We used a two-arm, parallel group, cluster randomized controlled trial to evaluate the effect on pain outcomes of introducing EPAT into cancer centers (Appendix, online only, list of study group members). The trial clusters were the inpatient units of regional cancer centers in the United Kingdom. In each center, the trial was conducted in two phases. In the first, pre–random assignment phase, 50 patients with cancer-related pain were enrolled, and their pain outcomes were measured after management in accordance with UC. Centers were then randomly assigned to either implement EPAT (EPAT centers) or to continue providing UC (UC centers). In the second, post–random assignment phase, an additional 50 patients were enrolled and their pain outcomes were measured.

Cancer centers were eligible to participate if they did not have an existing bedside pain management system, could recruit 100 patients in the required time frame, and did not anticipate organizational changes that might affect pain management policies. Within each center, patients were eligible to participate if they were adults ( ≥ 18 years of age) with active cancer and cancer-related pain, with a worst pain in the past 24 hours score (assessed within 24 hours of admission) of ≥ 4 on a scale of 0 to 10. The protocol (Data Supplement) lists complete inclusion and exclusion criteria.

The trial was approved by the Scotland A Research Ethics Committee and was overseen by a trial steering committee. Conduct of the trial in each center followed study guidance procedures (Data Supplement). All centers and participants provided written informed consent.

### Random Assignment and Masking

A database software algorithm, implemented by the Edinburgh Clinical Trials Unit, randomly allocated cancer centers to implement EPAT or continue providing UC in a 1:1 ratio using variable-size permuted blocks. Because of the cluster design, we were unable to mask cancer center clinicians, patients, or data collectors to intervention. However, the clinicians in the UC centers did not know the content of EPAT, and patients self-rated their pain and knew only that their cancer center was taking part in a pain study.

### Procedures

#### Patient enrollment.

All patients admitted to the participating cancer centers were given information about the study. A research nurse obtained patients’ verbal consent to eligibility screening. The nurse provided eligible patients with a further explanation of the study and obtained their written consent for participation. Enrollment was done independently of the clinical team.

#### Delivery of pain management.

The clinicians who delivered pain management (EPAT or UC) were oncology nurses, nursing care assistants, oncology trainee doctors, and senior oncologists. In UC centers, the clinical team managed patients’ pain according to their clinical judgment and existing local guidelines. In EPAT centers, the clinical team was provided with the EPAT (Data Supplement) and given brief (maximum, 1 hour) training in its use. EPAT was designed to address the aforementioned key barriers to effective pain management by prompting clinicians to systematically assess pain using simple questions that should not be shortened or paraphrased; to follow linked treatment algorithms, rather than broad guidelines, including instructions on opioid prescribing; and to regularly reassess pain and opioid-related adverse effects.

**Fig 1.**
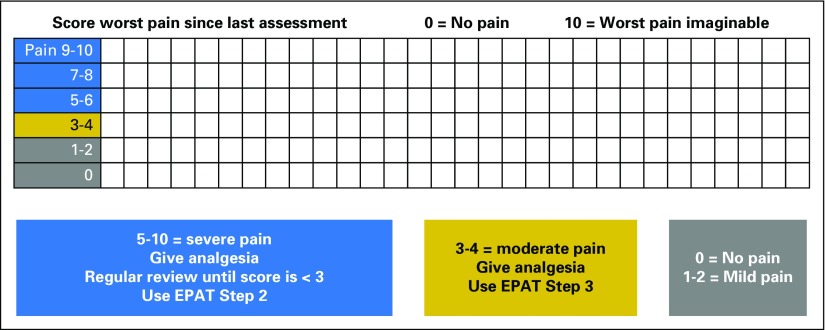
Edinburgh Pain Assessment and management Tool (EPAT) step 1 as it appears on the vital signs chart.

EPAT integrates pain assessment into routine care by its inclusion in the patient’s bedside chart. Clinicians are prompted to assess pain using a two-step procedure every time the patient’s vital signs are recorded. In step 1 (Fig 1), the patient is asked to rate his or her worst pain (since last being assessed) on a scale from 0 (no pain) to 10 (worst pain imaginable), and this score is recorded. The chart categorizes pain scores using the following color system: 0 to 2 is classified as gray, 3 to 4 as yellow, and 5 to 10 as blue. For patients with a yellow or blue score, the clinician is prompted to proceed to step 2 (Data Supplement), which explores the location and nature of the pain, exacerbating and relieving factors, and symptoms that may be caused by opioids. Flags linked to recorded responses then prompt the clinician to use the appropriate algorithm to guide prescribing. The chart also prompts reassessment of pain and opioid adverse effects 1 hour after administration of opioid medication.

### Outcomes

The primary trial outcome (measured at the cancer center level) was the change in the percentage of participants with a clinically significant improvement in pain. We defined a clinically significant improvement in pain as a reduction of ≥ 2 points in the severity of worst pain reported over the previous 24 hours measured between admission and reassessment (3 to 5 days after admission). Worst pain is an item on the Brief Pain Inventory Short Form (BPI-SF), which has 11 questions, each rated on a scale of 0 to 10, and is validated for use in the evaluation of cancer pain.^17^ Worst pain is the recommended outcome measure for trials, and a 2-point change on this item has been found to be meaningful to patients.^18-20^

The secondary trial outcomes (also measured as cancer center–level changes) were the percentage of participants with controlled pain (worst pain BPI-SF score < 4); the mean changes in BPI-SF scores (worst pain, pain subscale, pain interference subscale, and total score); the mean change in global distress in the past 24 hours using the National Comprehensive Cancer Network thermometer (0 to 10 thermometer, where 0 is no distress and 10 is extreme distress)^21^; the mean change in opioid adverse effects (using the mean of 0 to 10 scales for drowsiness, confusion, disorientation, shadows, vivid dreams, hallucinations, and muscle twitching; a modification of a previously used scale)^22^; the percentage of participants receiving good practice prescribing (rated based on the appropriateness of the medication[s] prescribed, along with how they were prescribed, compared with guidelines; Data Supplement); the percentage of participants readmitted to the cancer center with uncontrolled pain within 14 days of discharge; and in EPAT centers, satisfaction with the attention given to pain and the ease of use of EPAT rated on a 0 to 10 scale by patients and nurses, respectively.

Outcome data were collected from participants, medical records, and relevant staff, and as appropriate, by the research nurses. If participants had been discharged from the cancer center before the time of the follow-up assessment, the research nurse collected the outcome data by telephone.

### Statistical Analysis

We estimated that two treatment groups of nine cancer centers each, with 100 participants per cancer center (50 recruited in the first phase before random assignment of the cancer center, and 50 recruited in the second phase after random assignment) would give 80% power at the 5% significance level to detect a difference of at least 15% between the trial arms in the primary outcome. The main analysis was conducted (using SAS version 9.2 software; SAS Institute, Cary, NC) at the end of the trial, using an intent-to-treat principle that included all participants who provided usable outcome data.

Because there were, as planned, relatively few clusters (cancer centers) and little variability in the cluster size, we performed the analyses using summary measures.^23^ For each analysis, we calculated a single summary measure for each cancer center and compared the UC centers with the EPAT centers using a two-sample *t* test. Hence, the summary statistic for the primary outcome was the difference between the percentage of participants who achieved a clinically significant reduction in pain before random assignment (phase 1) and the percentage who achieved a clinically significant reduction in pain after random assignment (phase 2). For continuous outcome measures, the summary statistic was the difference in the mean score before and after random assignment.

In a prespecified sensitivity analysis, hierarchical patient-level analyses were performed using random-effects models. The results for the primary end point (achieving a reduction of ≥ 2 points in the severity of worst pain reported over the previous 24 hours) and a secondary end point (the magnitude of the reduction in the severity of worst pain reported over the previous 24 hours) are reported. In addition to the main analysis, we performed a post hoc sensitivity analysis of the primary outcome and of good practice prescribing excluding cancer centers that had been randomly assigned to EPAT but were unable to implement it.

## RESULTS

**Fig 2.**
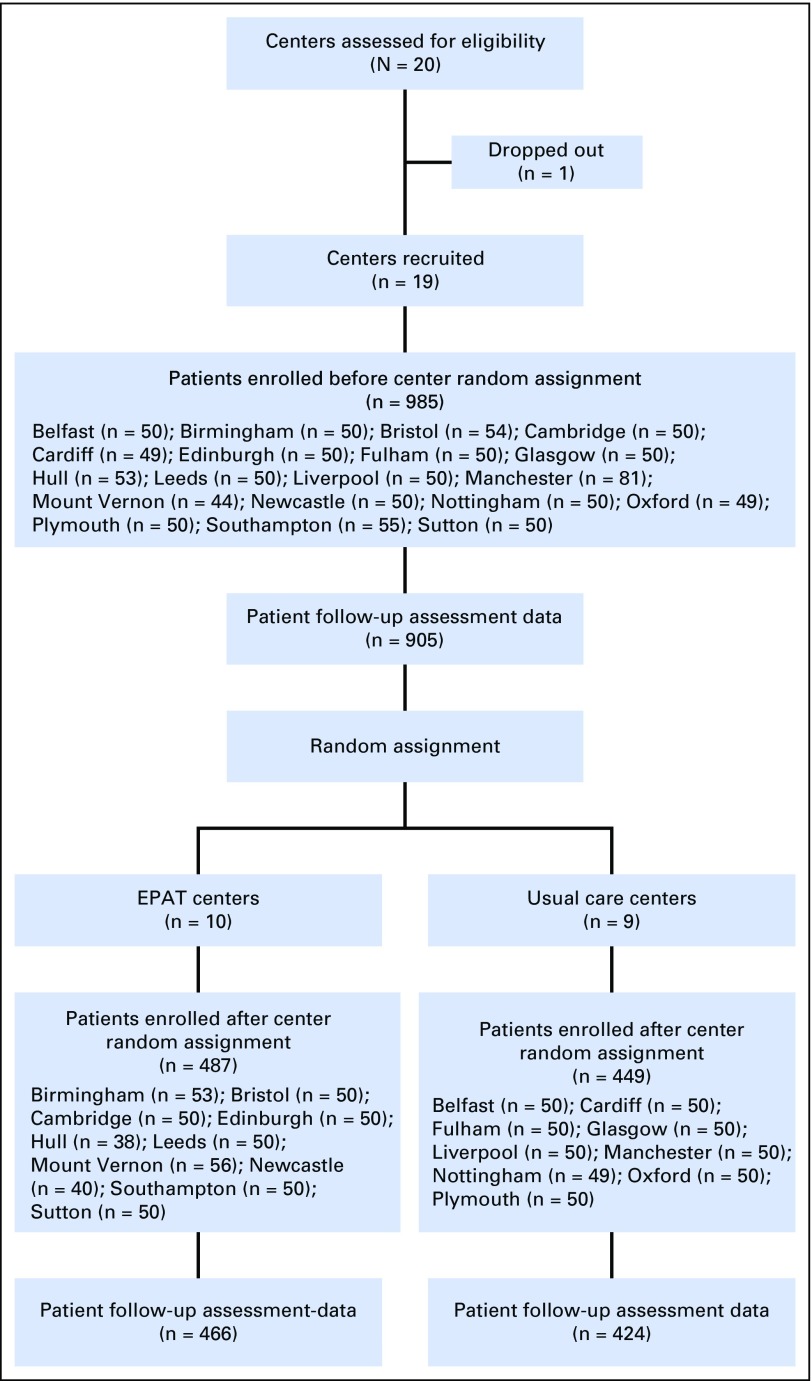
CONSORT diagram. EPAT, Edinburgh Pain Assessment and management Tool.

The United Kingdom has 40 cancer centers. We selected 20 centers from those eligible to participate, aiming for geographical spread and a range of center sizes. Each center took part in the trial for approximately 1 year (including both pre– and post–random assignment phases). Center participation was staggered and occurred between December 2007 and January 2013. One center dropped out before random assignment. Ten cancer centers were randomly assigned to implement EPAT, and nine were assigned to continue providing UC (Fig 2).

Of the 42,000 patients admitted to the cancer centers during the trial, 8,400 were assessed as having moderate or severe pain (worst pain in the past 24 hours of ≥ 4 on a 0 to 10 scale), and 1,921 patients were enrolled (985 before center random assignment and 936 after assignment; Data Supplement). The most common reason for excluding patients was that their admission was for planned chemotherapy and too brief to allow completion of the protocol. Only 2.7% of eligible patients (53 of 1,974 patients) declined to participate. Participants had a mean age of 60 years (range, 20 to 90 years), and 49% were female (Table 1). Patients had a variety of cancers, the most common of which were genitourinary (272 of 1,921 patients; 14.2%), GI (260 of 1,921 patients; 13.5%), breast (234 of 1,921 patients; 12%), and lung (220 of 1,921 patients; 11.5%). These characteristics were well balanced between EPAT and UC centers. We obtained outcome data from 93% of participants (1,795 of 1,921 patients). These data included 150 telephone follow-up assessments for patients who had been discharged from hospital (the number of telephone assessments was similar in both trial arms). The mean number of days from admission to the primary outcome assessment was 4 and was similar between trial arms.

**Table 1.**
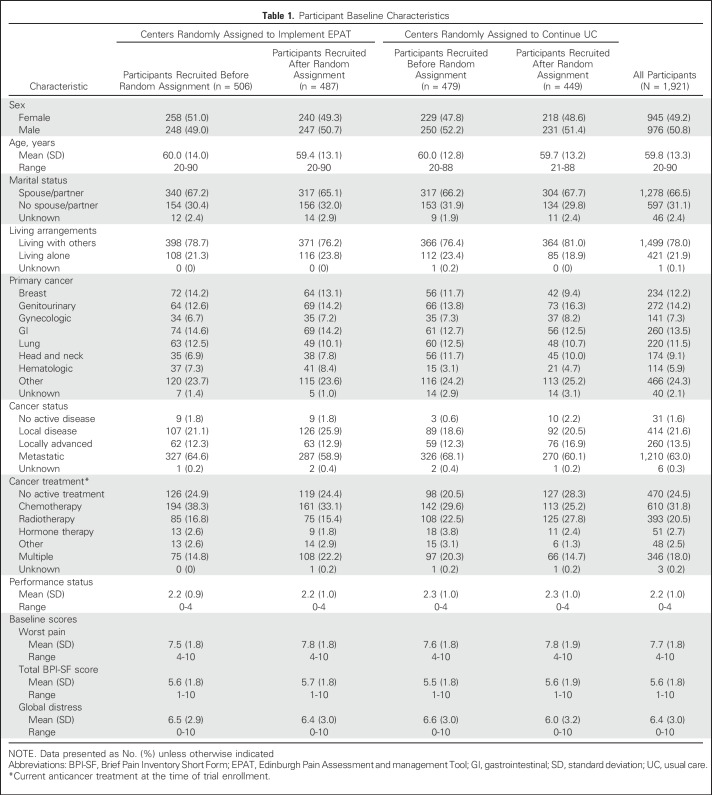
Participant Baseline Characteristics

**Fig 3.**
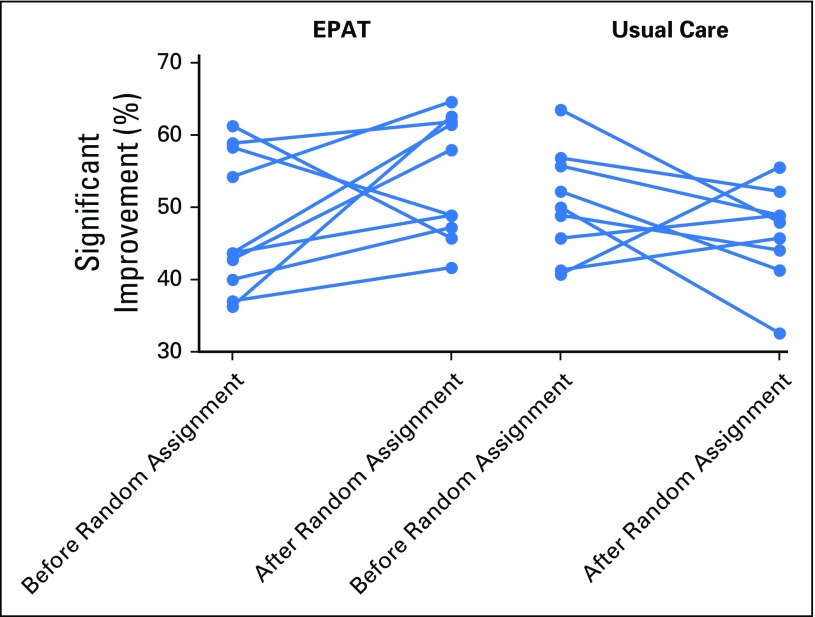
Plot of primary outcome by center. EPAT, Edinburgh Pain Assessment and management Tool.

Introducing EPAT into cancer centers had an effect on pain outcomes. The percentage of participants with a clinically significant improvement in pain increased after random assignment in eight of the 10 EPAT centers and in three of the nine UC centers (Fig 3). In EPAT centers, the mean percentage of participants with a clinically significant improvement in pain increased from 47.7% (before random assignment) to 54.1% (after random assignment), an absolute increase of 6.4%. In UC centers, the mean percentage of participants with a clinically significant improvement in pain decreased from 50.6% (before random assignment) to 46.4% (after random assignment), an absolute decrease of 4.2%. Thus, the absolute difference between trial arms was 10.7% (95% CI, 0.2% to 21.1%; *P* = .046). There was no difference in outcomes between participants assessed by telephone after discharge and those who were assessed as inpatients.

Two of the centers randomly assigned to implement EPAT were unable to do so because of unanticipated organizational changes. In these centers, the percentage of participants with a clinically significant improvement in pain actually decreased after random assignment (Fig 3). A post hoc sensitivity analysis that excluded these two centers found a larger difference between EPAT and UC (absolute difference, 15.4%; 95% CI, 5.8% to 25.0%; *P* = .004). The remaining eight centers that implemented EPAT used it in at least 90% of patient assessments as indicated by entries on the participants’ charts.

Regarding the trial’s secondary outcomes, EPAT centers had greater improvements in good practice prescribing (a difference that was larger when the two centers unable to implement EPAT were excluded) and greater changes in the mean worst pain item and in mean pain subscale scores. However, there were no statistically significant differences between EPAT and UC centers in the percentage of participants with controlled pain, the mean pain interference score, the mean total pain score, or the mean severity of global distress. There was also no difference between EPAT and UC centers in the percentage of patients who had received strong opioids (80%), in the mean total 24-hour oral morphine equivalent (10 mg), or importantly, in opioid-related adverse effects (Table 2). We were unable to analyze readmissions to hospital because the available data were inadequate. Participants who received EPAT reported (on a 0 to 10 scale) high satisfaction with the attention given to their pain (mean score, 8.6; standard deviation, 1.8), and nurses reported (on a 0 to 10 scale) moderate satisfaction with the ease of using EPAT (mean score, 6.4; standard deviation, 2.5).

**Table 2.**
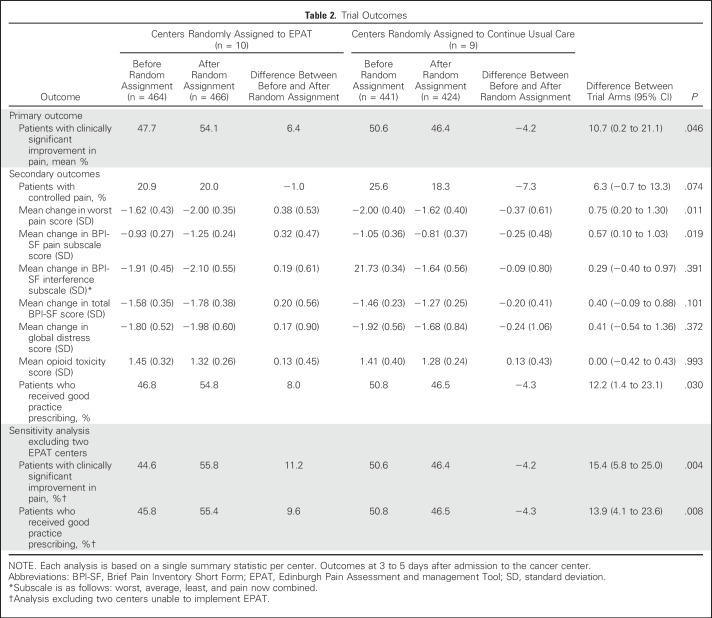
Trial Outcomes

From the prespecified patient-level sensitivity analysis of the primary end point, the estimated intracluster correlation coefficient was 0.004, and the estimated treatment effect was 11.6% (95% CI, 2.4% to 20.9%; *P* = .014). For the corresponding analysis of the magnitude of the reduction in the patients’ worst pain scores, the estimated intracluster correlation coefficient was 0. The estimated treatment effect was 0.82 (95% CI, 0.31 to 1.32; *P* = .002).

## DISCUSSION

The findings of this multicenter, cluster randomized trial indicate that a policy of integrating systematic pain assessment and management into routine cancer center care using a simple tool (EPAT) improves pain outcomes for patients with moderate or severe cancer-related pain. The difference between EPAT and UC centers in the percentage of patients with a clinically significant reduction in their worst pain was 10.7%. This difference increased to 15.4% when the two centers that had been unable to implement EPAT were excluded. It should be noted, however, that the 95% CI for the 10.7% difference is wide (0.2% to 21.1%).

Inspection of the results by cancer center revealed that the difference in the primary outcome between centers delivering EPAT and centers continuing UC reflected not only an improvement in pain management with EPAT (except in the two centers that failed to implement EPAT), but also a deterioration in pain management in most of the centers that continued UC. We did not find any differences in patient or cancer characteristics in the samples studied in each phase that could account for this. Therefore, we suggest that a likely explanation for the deterioration in outcome in some, but not all, of the UC centers in the post–random assignment period reflects a return to pretrial standards of pain management after improvement during the pre–random assignment phase of the trial. This short-term effect on clinicians’ behavior might be expected as a result of awareness that their center was participating in a study that monitored their patients’ pain scores.^24^ This effect would also be expected to decline over the many months of study in centers continuing with UC. The effect was not seen in centers implementing EPAT, which overall had better outcomes.

EPAT centers prescribed analgesics more appropriately (as defined in the Data Supplement) and not in higher doses. Although the concept of pain as the fifth vital sign has been criticized as a potential cause of increased opioid-related adverse effects, the use of EPAT did not increase opioid adverse effects.^25,26^ This absence of increased adverse effects despite better pain management may be because EPAT alerts clinicians to monitor adverse effects of pain treatment, as well as efficacy.

EPAT did not improve all of the secondary trial outcomes. The percentage of participants with controlled pain, the severity of general distress, and the degree to which pain interfered with activities did not differ between trial arms. The short duration of this study and inpatient setting arguably made it difficult to change these outcomes.

An additional and notable finding is that implementation of EPAT was hampered by organizational and leadership changes in two trial centers, which also subsequently had worse patient outcomes. This observation highlights the importance of organizational factors and leadership, as well as clinician education, in achieving positive changes in patient care.^27^ As expected, the patient-level sensitivity analyses yielded parameter estimates and CIs that were broadly similar to those obtained in the primary cluster-level analyses and with levels of statistical significance that were more extreme.

Previously published smaller studies have evaluated the effects of systematic management for cancer pain. Two randomized trials compared algorithm-based pain management by pain specialists, rather than oncology teams, with variable results.^28,29^ Two preliminary studies evaluated the integration of systematic pain management into the usual clinical care delivered by oncology teams with more promising findings.^30,31^ Finally, it is notable that published studies of integrated and systematic approaches to the management of other symptoms in patients with cancer, in particular depression, have also found these to be effective.^32-34^

The strengths of this trial include the participation of many cancer centers in the United Kingdom and negligible missing outcome data. However, the trial has limitations. First, the trial was carried out within one particular health care system (the United Kingdom National Health Service), although the issues associated with cancer pain assessment and management are similar in most developed countries. Second, we do not have information on participants’ longer term outcomes. Third, oncology teams and those collecting the outcome data from patients could not be masked to the treatment allocation.

In conclusion, this study is, to our knowledge, the first large randomized evaluation of the integration of systematic pain assessment and management into routine care of patients with cancer. The implementation of EPAT improved both prescribing practice and pain outcomes. Furthermore, it did not increase opioid-related adverse effects. This latter finding is important given concerns that the measurement of pain as a vital sign and linked opioid prescribing can be harmful.^14,15^ The findings of this trial add to the accumulating evidence for the efficacy of more integrated and systematic approaches to symptom management in patients with cancer.

## The Edinburgh Pain Assessment and Management Tool Study Group

Debra Gordon, Moira Ross, Lucy Norris, Eleanor Clausen, Elaine Boland, Sara Booth, Alison Coakley, Mary Comiskey, Andrew Davies, Carol Davis, Jane Edgecombe, Karen Forbes, Fiona Hicks,† Jamal Humaira, Jane Maher, Wendy Makin, Mary Nugent, Nikki Pease, Allan Price, Julia Riley, Joy Ross, Kirsten Saharia, John Speakman, John Walley, Bee Wee, Andrew Wilcock, Pauline Wilkinson, Teresa Young, and Andrew Walker.

^†^Deceased September 2015.
